# Dietary Patterns and Their Association with Metabolic Syndrome and Their Components in Middle-Class Adults from Damascus, Syria: A Cross-Sectional Study

**DOI:** 10.1155/2022/5621701

**Published:** 2022-03-24

**Authors:** Malda Atasi, Ashuin Kammar-García, Rafael Almendra-Pegueros, Addi Rhode Navarro-Cruz

**Affiliations:** ^1^Syrian Private University, Damascus, Syria; ^2^Dirección de Investigación, Instituto Nacional de Geriatría, Mexico City, Mexico; ^3^Sección de Estudios de Posgrado e Investigación, Escuela Superior de Medicina, Instituto Politécnico Nacional, Mexico City, Mexico; ^4^Laboratorio de Investigación Traslacional en Farmacología, Facultad de Medicina, UASLP, San Luis Potosí, Mexico; ^5^Escuela de Ciencias de la Salud, Universidad del Valle de México, San Luis Potosí, Mexico; ^6^Department of Biochemistry and Foods, Faculty of Chemical Sciences, Benemérita Universidad Autónoma de Puebla, Puebla City, Mexico

## Abstract

Prior to the 2016 crisis in Syria, a study conducted in Aleppo found the prevalence of metabolic syndrome to be 39.6%, which is known to be favoured by age and poor lifestyle (including physical inactivity and the consumption of hypercaloric foods, rich in saturated fats, concentrated carbohydrates, and salt), so the objective of this study was to identify the association of different dietary patterns with metabolic syndrome and their components. A cross-sectional analytical study was carried out in 104 adults aged 40 to 65 years who did not suffer from previous diseases. The sample was chosen from middle-class citizens of the city of Damascus who were contacted by telephone; they were explained about the study, the information that would be collected, and the studies that should be carried out in the clinical analysis laboratory of the Private University of Syria. A nutritional and food study was carried out using previously validated forms containing 62 items in which the food intake of the participants was studied. We apply principal component analysis and factor analysis to detect nutritional components and dietary patterns. Dietary pattern 3 (foods with simple carbohydrates and saturated fat) increased glucose levels, while dietary patterns 1 (high intake of calories, protein, and saturated fat) and 5 (fast food) increased serum triglyceride levels. In addition, pattern 1 (carbonated beverages, grains, chicken, and meat) was associated with elevated LDL cholesterol levels and the presence of the metabolic syndrome. The study findings suggest that the presence of metabolic syndrome and its components are associated with dietary patterns high in calories, protein, simple carbohydrates, and saturated fat.

## 1. Introduction

The Syrian Arab Republic is in the Middle East, on the coast of the Mediterranean Sea, it has an approximate population of 16.5 million inhabitants, and the main cities are Aleppo, Homs, and Damascus, the latter being the capital. The country has shown limited progress towards achieving diet-related noncommunicable disease (NCD) targets and has shown no progress towards achieving the obesity target, with an estimated 38.3% of adult women (over 18 years) and 24.1% of adult men living with obesity. The prevalence of obesity in Syria is higher than the regional average of 10.3% for women and 7.5% for men [1]. In 2016, according to the WHO, the prevalence of diabetes in Syria was 11.9%, the overweight 55%, and the obesity 21.6% [2], without policies, guidelines, or surveillance, and although there are national guidelines/protocols/norms against evidence-based diabetes, they do not apply, and the projection of diabetes in men and women for 2019 was 17.8% for women and 16.4% for men [3].

Before the crisis in Syria, a study conducted in Aleppo determined the prevalence of MetS at 39.6%. The most prevalent component was arterial hypertension (56.6%), followed by central obesity (51.4%). The high prevalence of MetS and its components emphasizes the burden of cardiovascular disease among adults in this population [4].

Metabolic syndrome (MetS) is the commonly observed group of visceral adiposity, endothelial dysfunction, atherogenic dyslipidemia, elevated blood pressure, insulin resistance, hypercoagulable state, and chronic stress that together increase the risk of developing cardiovascular disease, type 2 diabetes mellitus, and all-cause mortality [5–7].

For many years, the relationship of the etiopathogenesis of certain diseases with the consumption of food or certain nutrients was evaluated; however, this type of study has many limitations, such as the fact that it is a challenge to detect and verify the true associations between disease and individual nutrients. There are complex interactions between nutrients in consumed foods that do not show the interactive and synergistic effects of different foods and nutrients in a diet, making it difficult to detect an independent influence [8–10].

A better approximation has been achieved with the dietary patterns, which allows us to examine the potential combined effect of the entire diet on human health and can be determined by *a priori* or *a posteriori* methods [10]. The *a priori* approach involves the estimation of a dietary index/score based on the prior association of foods and diseases, for example, the Mediterranean diet score commonly applied in epidemiological studies and used as an *a priori* approach in studies that seek the association between the Mediterranean diet and noncommunicable diseases, and it has been found that adherence to the Mediterranean diet is associated with a lower probability of having MetS [11]; however, in many Mediterranean countries, the prevalence of MetS is not low; for example, in Turkey, Tunisia, Palestine, Iraq, Saudi Arabia, Jordan, or Iran, the prevalence of MetS exceeds 30% [12–18]. On the other hand, the *a posteriori* approach is based on statistical methods, such as factor analysis, principal components analysis, and cluster analysis, and although the results of many studies that have used this approach might seem contradictory, the accumulated evidence suggests that there is a relationship between dietary patterns and the risk of MetS [9, 10, 19–21]. One of the priorities in Public Health is the identification of eating patterns that allow establishing associations with health [22]; the purpose of this study is to analyze the different dietary patterns of a sample of Syrian adults and evaluate the association between the observed eating patterns with the MetS and their components.

## 2. Methods

### 2.1. Study Design and Participants

A cross-sectional analytical study was conducted. Convenience sampling was employed and was chosen from middle-class citizens of the city of Damascus between the ages of 40 and 65 who had not been diagnosed with previous illnesses and who wanted to attend the Clinical Analysis Laboratory of the Syrian Private University for clinical analysis. The project was presented to those interested in participating, and their informed consent was requested. The sample size was calculated to estimate the prevalence of metabolic syndrome considering the prevalence of 39.6% reported by Ramadan et al. [4]. The calculated sample size was 369 subjects, considering a precision of 5% and a confidence interval of 95%. Of the total of 370 possible participants interviewed, only 130 met the inclusion criteria, and of these, only 106 people completed the process of taking a blood sample for analysis and the information required in the questionnaire. All data were collected during the period from November 2016 to February 2017.

In the absence of a bioethics committee constituted in the Private University of Syria, the study protocol was approved by the scientific research and the Departmental Board of Biochemistry-Food of the Faculty of Chemical Sciences of the BUAP in Mexico. This study was conducted in accordance with the guidelines prescribed by the Declaration of Helsinki.

### 2.2. Nutrition and Food Assessment

The nutritional and food study was completed using a previously validated food frequency questionnaire containing 62 items [23], in which the food intake of the participants was studied, and specific measures were developed for each of these items, placing the size of the food next to each type of food, the ration, or quantities used in homemade measures (cup, teaspoon, sprinkle, etc.). The results were analyzed using the Alimentador software (online application for calculating personalized diets). Foods were grouped according to the similarity of their ingredients, their nutritional profile, and their culinary use within 25 groups of foods frequently consumed in the Syrian population (Supplementary Table S1), which was previously described [23]; the amount in grams of consumption was determined for each food group.

### 2.3. Anthropometric, Clinical, and Biochemical Data

Weight, height, and waist circumference were taken using specific techniques and methods by trained researchers, and blood pressure was measured with a Mercury manometer (Alpk2 500V Aneroid Sphygmomanometer, Japan) after setup and the participant at rest for five minutes. The nutritional status was classified as normal weight (BMI < 25 kg/m^2^), overweight (BMI = 25–29.9 kg/m^2^), and obesity (BMI ≥ 30 kg/m^2^).

After a 12-hour fast, the blood samples were collected and sent immediately to the Laboratory of Clinical Analysis of the Faculty of Pharmacy of the Private University of Syria. Serum was separated by centrifugation, and triglyceride, HDL-C, cholesterol, and glucose levels were measured by an enzymatic spectrophotometric technique using the Siemens ADVIA 1800 Chemistry Analyzer (Bellport, NY).

### 2.4. Sociodemographic Data

The demographic data of the participants were obtained through a questionnaire applied after anthropometric, clinical, and laboratory measurements, the data obtained were smoking (defined as consumption of any tobacco product in the last 6 months), history of clinical obesity of parents, educational level (primary, secondary, high school, institute, university, and higher education), marital status, and physical activity. The physical activity was determined using the short version of the International Physical Activity Questionnaire (IPAQ) [24], with four general questions about the type and duration of exercise carried out during the last 7 days, and they were recorded as sedentary when the participant did not obtain a score within the moderate and intense categories: moderate: when vigorous activity was presented for 3 days or more with a minimum of 20 min or a walk of 5 days or more with a minimum of 30 min; intense: when any moderate or high-intensity activity that added 3000 METs (minutes/week) was reported 7 days a week or when vigorous activity was performed for 3 days a week, adding up to 1500 MET (minutes/week).

### 2.5. Metabolic Syndrome

The criterion of the Adult Treatment Panel III (ATP-III 2001) [25] was used, which considers that people have metabolic syndrome if they have 3 or more of the following criteria: waist measurement >102 cm in men and >88 cm in women; 150 mg/dL triglycerides; HDL cholesterol <40 mg/dL in men and <50 mg/dL in women; blood pressure >130/85 mmHg; fasting glucose> 110 mg/dL.

### 2.6. Statistical Analysis

Data are presented descriptively as mean and standard deviation (SD) for quantitative variables, while qualitative variables are presented as frequencies and percentages. All the variables were explored by means of skewness and kurtosis to determine if the distributions of each variable were the most like the normal distribution. Comparisons of nutrient intake between the different numbers of metabolic alterations were carried out by means of a one-way ANOVA. Dunnett's post hoc test was applied to compare the means of the various groups with the group of patients without any metabolic alteration. Data were summarized as mean ± standard error (SE). A principal component analysis was applied to reduce the dimensions of the dietary intake variables. The coefficients of the principal components that had eigenvalues greater than 1 were extracted. Subsequently, a factorial analysis was carried out to determine the different dietary patterns from the consumption of the different food groups, the correlation matrix was made, which was analyzed with the Bartlett sphericity test, and the Kaiser–Meyer–Olkin statistic was calculated as an assumption for the application of exploratory factor analysis (FA). The charges were extracted from the matrix by means of the varimax technique. From the extraction of the main components of nutritional intake and of the factors of dietary patterns, linear regression models and logistic regression models were applied, and linear regression models were performed to determine a relationship between nutritional intake and patterns. With the linear increases with the metabolic parameters, the logistic regression models were used to estimate the strength of association between the components of nutritional intake or eating patterns (independent variables) with the various metabolic alterations and metabolic syndrome (dependent variables). The results of the linear regression models were summarized as *β* coefficients and the logistic regression models as odds ratio (OR) and 95% confidence intervals (95% CI). In both models, the independent variable was introduced quantitatively if there is a reference value. All regression models were adjusted for sex, age, and nutritional status. The assumptions of all models were verified by residual analysis. No data imputations of any kind were made.

A two-sided *p* < 0.05 was considered statistical significance. All the analyzes were carried out with the statistical package SPSS v.21 and the R software v.3.6.2.

## 3. Results

During the November 2016–February 2017 period of the 350 possible participants interviewed, only 130 met the inclusion criteria, and of these, only 104 participants were selected for having complete data on blood sampling, 55.8% (*n* = 58) corresponded to the female sex, and the mean age was 47.6 (SD: 5.7). 72.5% of the participants reported being married, and 66.3% had a university education and higher education. 31.7% of the sample reported smoking. As can be seen in Table 1, the frequency of overweight/obesity was 60.6% (BMI 36.35; SD: 4.11 kg/m^2^) and 69.6% with a history of parents with obesity. 22.5% were diagnosed with metabolic syndrome, being higher in men (28.3% versus 17.2%). Of the components that make up the diagnosis of metabolic syndrome, the most altered were elevated waist circumference (61.5%, media: 97.35, and SD: 15.54 cm), elevated total cholesterol (37.5%, media: 187.68, and SD: 6.05 mg/dL), and decreased HDL cholesterol (33.7%, media: 52.48, and SD: 14.61 mg/dL).

The average energy consumption in the sample was 2556.33 (SD: 931.4) Kcal, 309.39 (SD: 126.08) g of carbohydrates, 86.77 (SD: 34.58) g of protein, and 117.37 (SD: 58.35) g of lipids. Fiber intake was 21.40 (SD: 10.63) g, and dietary cholesterol was 299.64 (SD: 18.76). The intake of another critical nutrient is presented in Table 2. Of the 25 food groups, the sample only presents the intake of 21 groups as shown in Table 3; the groups that were not reported by the participants were breakfast cereals (regular corn flakes), low-fat dairy products (half-skimmed milk, low-fat cheese, and low-fat yogurt), full-fat dairy products (whole milk, whole-fat cheese, labneh (strained yogurt), and whole-fat yogurt), and legumes (all kinds of legumes like beans, lentils, chickpeas, fava beans, and peas).

Table 4 shows the comparison of nutrient and food consumption by the number of metabolic syndrome components. Those who presented three metabolic syndrome components reported a higher intake of proteins (92.2 ± 4.9 versus 75.9 ± 10 g, *p*=0.03), lipids (133.5 ± 10 versus 91.7 ± 10.9 g, *p*=0.01), monounsaturated fatty acids (57.5 ± 5.1 versus 38.1 ± 5.4 mg, *p*=0.01), polyunsaturated fatty acids (24.3 ± 2.4 versus 14.7 ± 2.3 mg, *p*=0.02), and vitamin E (19.4 ± 1.9 versus 12.1 ± 1.8 mg, *p*=0.04) that those without any metabolic syndrome component. In the case of folic acid, the behavior was the opposite (584.2 ± 42.4 versus 640.0 ± 96.7 mg, *p*=0.03).

A principal component analysis (PCA) was applied to reduce the dimensions of the variables of nutrients consumed, 5 main components were obtained from the eigenvalues (component 1 : 10.77, component 2 : 1.94, component 3 : 1.52, component 4 : 1.36, and component 5 : 1.07), and these 5 components explain 72.5% of the total variance. The coefficients of each component are observed in Table 5; only the coefficients of the first 5 principal components are shown.

The factorial analysis showed the presence of 5 dietary patterns. Pattern 1 is composed of foods with high caloric-protein intake and saturated fat. Dietary pattern 2 comprises foods of traditional consumption in the Syrian diet. Pattern 3 comprises foods with a high contribution of simple carbohydrates and saturated fat. Pattern 4 comprises foods of traditional consumption in the Syrian diet with a predominance of mono and polyunsaturated fatty acids. Pattern 5 is formed by fast food with a high predominance of energy and sodium. Of the nutrients that make up component 1, protein consumption showed a trend associated with metabolic syndrome, odds ratio (OR): 1.012, 95% CI: 0.99–1.02, and*p*=0.08. The coefficients of each component are observed in Table 5, and the loads presented in each dietary pattern are shown in Table 6.

**Figure 1:**
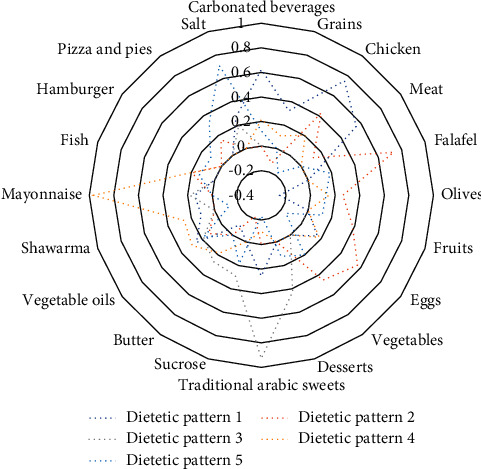
Radar graph of factor loadings for each dietary pattern. Dietetic pattern 1 is composed of foods with high caloric-protein intake and saturated fat. Dietary pattern 2 comprises foods of traditional consumption in the Syrian diet. Pattern 3 is composed of foods with a high contribution of simple carbohydrates and saturated fat. Pattern 4 is composed of foods of traditional consumption in the Syrian diet with a predominance of mono- and polyunsaturated fatty acids. Pattern 5 is formed by fast food with a high predominance of energy and sodium.

Table 7 shows the linear regression analysis between metabolic parameters and main components of nutritional intake adjusted for sex and age.  Figure 1 illustrates factor loadings that characterized each dietary pattern. The linear regression analysis showed that the increase in energy intake (*β*: 0.001, SE: 0.0001, and *p*=0.04), proteins (*β*: 0.026, SE: 0.012, and *p*=0.03), lipids (*β*: 0.020, SE: 0.007, and *p*=0.04), and saturated fatty acids (*β*: 0.019, SE: 0.009, and *p*=0.03) was associated with the linear increase in BMI. Consumption of component 1 nutrients (energy, fiber, proteins, lipids, total cholesterol, vitamin A, D, and B_12_, and potassium) was associated with systolic blood pressure (*β*: 0.79 and *p*=0.04) and with diastolic blood pressure (*β*: 0.64 and *p*=0.02). Eating pattern three, foods with simple carbohydrates and saturated fat, increased glucose levels (*β*: 58.06 and *p*=0.01), while dietary patterns 1 (high intake of calories, protein, and saturated fat) and 5 (fast food) increased serum triglyceride levels, as follows: dietary pattern 1: *β*: 130.92 and *p*=0.001; dietary pattern 5: *β*: 94.2 and *p*=0.05.

Table 8 shows the risk of metabolic syndrome components based on the components of nutritional intake or dietary patterns adjusted by sex, age, and nutritional status. Component 2 (carbohydrates, sugars, and vitamin K) was negatively associated with hypertension, while component 5 (sodium and vitamin B_6_) was positively associated with low HDL levels. Regarding dietary patterns, pattern 1 (carbonated beverages, grains, chicken, and meat) was associated with elevated levels of LDL cholesterol and in general with metabolic syndrome, while dietary pattern 3 (desserts, traditional Arabic sweets, sucrose, and butter) was associated with hyperglycemia but not with metabolic syndrome.

## 4. Discussion

Syria, like most developing countries, faces a “double burden” of noncommunicable diseases [26]. The prevalence of obesity in adults in Syria in 2016 was 28%. Syria ranks ninth in the ranking of deaths from coronary heart disease, with a death rate of 298.16 per 100,000. The national prevalence of diabetes in Syria, also in 2016, was 7.4% [26]. These chronic diseases may not have been treated since the conflict began due to the loss of health infrastructure, although recently the Syrian government provided universal healthcare and wealthy Syrians were also able to access private universities and health clinics, and although life expectancy at birth is 63.8 years, healthy life expectancy is 55.8 years [27]; despite this, few studies have been conducted in Syria to determine the prevalence of metabolic syndrome and its components. Research on metabolic syndrome has become critical for the prevention of type 2 diabetes, as it has been associated with a moderate risk of developing cardiovascular disease and a considerable risk of developing type 2 diabetes [28–30].

In a study carried out in Aleppo, the most populous city in Syria, it was found that the prevalence of metabolic syndrome was 39.6% with no difference between men and women [4], contrasting with 22.5% detected in our study, carried out in the city of Damascus, which could be explained since there are reports that the prevalence is higher in large cities, and even the prevalence of the metabolic syndrome is usually higher in the urban area population of some developing countries than in their Western counterparts [31, 32]; however, despite having a lower prevalence of MetS, the levels of overweight plus obesity are very high, 60.6%, and it has been observed that the higher the rates of overweight and obesity, the higher the prevalence of MetS [33]. Unlike most studies [34], we found that the prevalence was higher among men than that among women (28.3% versus 17.2%), perhaps due to the higher prevalence of overweight and obesity among men, as well as a higher prevalence of abdominal obesity, hypertension, and other factors that could condition the appearance of MetS [35]. Some studies report that one of the most prevalent components in the metabolic syndrome is low HDL-C [36, 37]; in our study, we found that central obesity was the most prevalent component (61.5%), followed by elevated total cholesterol (37.5%) and reduced HDL cholesterol (33.7%); however, it should not be forgotten that the risk associated with metabolic syndrome does not exceed its components and that successful management must address all the factors involved [38].

Syria is considered to be a country in transition; the average energy consumption obtained of 2,556.33, SD: 931.4 Kcal, is not as high as the average consumption that has been reported for these types of countries [39]; however, when the average consumption of macronutrients is observed, it can be observed that the consumption of lipids is higher than 40% of the total caloric intake with high consumption of cholesterol, which could be affecting the high levels of overweight and obesity observed among the population in this study, as there is convincing evidence that consuming high levels of energy-rich foods, such as foods high in fat and sugar, promotes obesity [40]. Among the participants who presented three metabolic syndrome components, a higher intake of proteins, lipids, monounsaturated fatty acids, polyunsaturated fatty acids, and vitamin E was reported compared to those without any component. In the case of folic acid, the behavior was the opposite, which is consistent with studies in which it has been suggested that a higher intake of folate may be associated with a lower MetS score and that the early correction of the levels of folic acid could help prevent cardiovascular complications in patients with MetS [41].

Methods for assessing associations of dietary intake and food groups with MetS are diverse; however, more recently, it has been proposed that dietary patterns are perhaps more predictive of chronic disease risk than consumption of individual foods or nutrients and can provide valuable information on the diet-disease association [42]; this is because people do not consume isolated nutrients but rather, according to their culture, consume a variety of dishes, so the evaluation would be more appropriate of the combined effect of nutrients by creating nutrient patterns to assess the effects of diet on disease [42].

Although MetS and its prevalence, as well as its main components, have been studied in the Syrian population, especially adolescents, there is practically no information on the associations between the main dietary patterns and the risk of MetS in Syrian adults. Therefore, due to the similarity of the Syrian diet to the Lebanese diet, we used the 25 frequently consumed food groups previously described by Naja et al. [23].

We identified five nutrient patterns and five dietary patterns. Most of the factors have been identified in previous studies [23, 40, 42]. In component 1, the highest nutrient load was energy, fiber, protein, cholesterol, fat, retinol, vitamin D, vitamin B12, and potassium, typical of a basic pattern of animal consumption. In component 2, the nutrients were carbohydrates, sugars, and vitamin K, typical of a starch-based pattern. In component 3, nutrients were MUFA, PUFA, AGS, and vitamin E, typical of a high-fat consumption base. In component 4, nutrient was folic acid, typical of a consumption base rich in vegetables. In component 5, nutrients were sodium and vitamin B6, typical of a consumption base of ultraprocessed foods.

Within component 1, protein consumption showed a trend associated with MetS, and although some studies show that protein consumption can promote weight loss and decrease the prevalence of MetS, it is very important to consider the context of the dietary pattern, since when it is associated with a plant-based diet, it can be protective, but in the context of the consumption of red meat and high amounts of fat, it is not [43]. Regarding the relationship with BMI, our results agree with studies that have verified an association between the intake of animal proteins with an increased risk of obesity [44], and although it is unlikely to be the sole contributor to obesity, the higher intake of animal protein among overweight/obese people could be attributed to the intake of red meat and its products which are energy-dense foods, and this could explain its association with the BMI [45].

Also, this component 1 was associated with systolic and diastolic blood pressure and BMI. Although the reported effects of dietary protein on hypertension are inconsistent across the literature, findings of higher blood pressure among meat eaters compared to vegetarians suggest that high dietary protein intake may be detrimental to blood pressure, even though the biological mechanism by which dietary protein influences the risk of hypertension and the variable effects of plant and animal protein intake remain unclear [46].

The factorial analysis evidenced 5 dietary patterns, which seemed to capture the nutrient intake patterns most appropriately: pattern 1: foods with a high caloric-protein intake and saturated fats that could be called the Western pattern, pattern 2: foods of traditional consumption in the Syrian diet, pattern 3: foods with a high carbohydrate intake simple and saturated fats, pattern 4: traditionally consumed foods in the Syrian diet with a predominance of monounsaturated and polyunsaturated fatty acids, and pattern 5: fast food with a high predominance of energy and sodium.

In dietary pattern 1, which we call the Western pattern, there is high consumption of carbonated beverages that provide a large amount of sugar, grains, red meat, and chicken. High consumption of sugary beverages has been independently associated with MetS. According to a study conducted in middle-aged adults, the consumption of ≥1 soda per day was associated with an increased likelihood of developing metabolic syndrome, obesity, increased waist circumference, impaired fasting glucose, and higher blood pressure [47]; in our case, this dietary pattern was mainly associated with hypertriglyceridemia.

Diet pattern 3, characterized by high consumption of simple carbohydrates and saturated fats, increased blood glucose levels and was distinguished by high consumption of Arab desserts and sweets that, although previously made with honey, today are made mainly with high fructose syrups or corn syrups, which makes them products with high glycemic index and the corresponding risk of MetS [23].

In dietary pattern 5, determined mainly by high consumption of foods with high energy density and sodium that we call fast food, the main foods were pizzas, hamburgers, pies, and nuts and were associated, like dietary pattern 1, with hypertriglyceridemia. Excessive consumption of energy-rich foods is likely to contribute to excessive energy intake, which, in turn, is linked to a higher prevalence of overweight/obesity and time-related chronic diseases. A diet with a positive energy balance and high-fat content or a high glycemic index content such as dietary pattern 5 may be a secondary cause and a contributor to hypertriglyceridemia [47].

Improving vitamin K status can improve cardiovascular health by reducing arterial stiffness and improving blood pressure [48], and when the risk of metabolic alterations is adjusted for sex, age, and nutritional status, it is observed that component 2 (carbohydrates, sugars, and vitamin K) was negatively associated with hypertension. Although it has been reported that changes in dietary sodium intake in the range of 50 to 150 mmol/day do not affect blood lipid concentrations [49], component 5 (sodium and vitamin B6) was positively associated with low HDL levels. Dietary pattern 1 (Western pattern) was associated with elevated LDL cholesterol levels and in general with MetS, while dietary pattern 3 (desserts, traditional Arabic sweets, sucrose, and butter) was associated with hyperglycemia but not with MetS.

Diet patterns 2 and 4, with consumption of traditional Syrian foods (eggplant, zucchini, cabbage, cauliflower, grape leaves, lentils, pickled turnips, cucumbers, tomatoes, sesame seeds, rice, chickpeas, broad beans, olive oil, lemon juice, mint, pistachios, garlic, lamb and sheep, honey, and fruits), were not associated with MetS and its metabolic abnormalities. The traditional Syrian pattern could be considered a Mediterranean pattern, but Mediterranean diets differ greatly between the countries of the Mediterranean basin. However, energy-rich foods such as traditional sweets are consumed a lot, so moderation in their consumption should be recommended.

Although the optimal dietary pattern to reduce the extent of metabolic syndrome (MetS) is not yet well established, the high-protein diet pattern had a direct effect on the manifestation of metabolic syndrome, as it was correlated with waist circumference, high blood pressure, BMI, and blood glucose levels, as well as high consumption of vegetable oils. Smoking, marriage, and having obese parents showed a positive effect on the manifestation of this syndrome. Therefore, it is recommended to avoid a high-protein diet, which does not exceed the suggested serving sizes, cook with a dusting of vegetable oils, try to eat at home, and practice moderate physical activity.

The main limitations of our study are the size of the sample and the possible inaccuracy of the dietary information; in addition to the fact that it is a cross-sectional study, causal inference is excluded. Since nutrient intake was assessed using an FFQ, some degree of measurement error is unavoidable; however, validation studies have generally been accepted as indicative of the ability of the FFQ to adequately classify people based on nutrient intake; also, it is not possible to completely exclude the effect of unmeasured sociodemographic factors such as income economic or food security, and also, we did not consider lifestyle factors such as quality and hours of sleep on the other hand, a limitation of this study is the difficulty in interpreting the results of the regression analyzes because the variables were introduced into the model quantitatively, but each of the main components or dietary factors do not have units. The interpretation of the magnitudes of effect sizes cannot be interpreted. The results of the regression analysis give information on the magnitude of the increase in probability due to the increase in the independent variable, that is, the amount of nutrients or dietary pattern.

Additionally, the use of *a posteriori* derived dietary patterns involves subjective decisions that may not reproduce the study findings, which would limit the comparability of our study. *A posteriori* approaches, such as factor analysis, rely on statistical methods, and although accumulating evidence suggests a relationship between post hoc dietary patterns and MetS risk, findings vary widely between studies. Some studies report that dietary patterns characterized by a high consumption of vegetables, fruits, and fish are inversely associated with MetS; conversely, dietary patterns characterized by a high consumption of red meat, processed meat, refined grains, alcohol, and fried foods are associated with a higher risk of MetS, and there are even studies that report that there is no significant association between dietary patterns characterized by meat and alcohol with MetS [50, 51].

## Figures and Tables

**Table 1:** Social, clinical, and anthropometric characteristics of the study sample.

	Total (*n* = 104)		Women (*n* = 58)		Men (*n* = 46)	
	Frequency	%	Frequency	%	Frequency	%
Sedentary activity	26	25.0	10	17.2	16	34.8
Metabolic syndrome	23	22.1	10	17.2	13	28.3
Marital status	75	72.1	33	56.9	42	91.3
*Educational level*						
Elementary	1	1.0	1	1.7	0	0.0
Secondary	5	4.8	2	3.4	3	6.5
High school	10	9.6	5	8.6	5	10.9
Institute	19	18.3	13	22.4	6	13.0
University and higher education	69	66.3	37	63.8	32	69.6
Parents with obesity	72	69.2	39	67.2	33	71.7
Smoker	33	31.7	15	25.9	18	39.1
Hyperglycemia	26	25.0	9	15.5	17	37.0
Hypertriglyceridemia	26	25.0	13	22.4	13	28.3
Hypercholesterolemia	39	37.5	21	36.2	18	39.1
High LDL	19	18.3	10	17.2	9	19.6
Obesity	22	21.2	9	15.5	13	28.3
Overweight	63	60.6	29	50.0	34	73.9
High DBP	16	15.4	6	10.3	10	21.7
High SBP	8	7.7	3	5.2	5	10.9
Low HDL	35	33.7	18	31.0	17	37.0
Large waist circumference	64	61.5	39	67.2	25	54.3
Hypertension	18	17.3	7	12.1	11	23.9
	Mean	SD	Mean	SD	Mean	SD
Glucose	92.47	30.12	85.22	16.68	101.60	39.63
Triglycerides	117.42	62.73	107.11	58.46	130.40	66.10
Cholesterol	187.68	36.05	186.71	34.27	188.90	38.53
HDL	52.48	14.61	57.23	14.36	46.48	12.71
LDL	111.94	39.31	111.00	35.38	113.11	44.14
Height	165.26	18.49	158.06	21.48	174.33	6.89
Weight	78.05	22.19	73.38	26.12	83.92	14.13
BMI	26.35	4.11	25.38	3.93	27.56	4.04
Waist circumference	97.35	12.54	92.74	12.47	103.15	10.06
DBP	76.97	9.28	75.17	9.22	79.24	8.94
SBP	119.90	12.88	116.72	13.03	123.91	11.64

DBP: diastolic blood pressure; SBP systolic blood pressure; BMI: body mass index; SD: standard deviation.

**Table 2:** Dietary intake of the study sample.

	Total (*n* = 104)		Women (*n* = 58)		Men (*n* = 46)	
	Mean	SD	Mean	SD	Mean	SD
Energy, Kcal	2556.63	941.34	2322.13	893.17	2852.28	926.02
Carbohydrates, g	309.39	126.08	283.93	133.12	341.50	109.76
Fiber, g	21.40	10.63	20.08	10.38	23.07	10.81
Protein, g	86.77	34.58	78.03	34.02	97.78	32.38
Dietary cholesterol, mg	299.64	158.76	285.00	156.48	318.11	161.40
Fat, g	117.37	58.35	106.61	53.94	130.93	61.39
MUFA, g	50.75	28.60	45.78	24.70	57.00	32.06
PUFA, g	21.63	14.17	19.11	11.42	24.80	16.60
SFA, g	31.03	42.75	32.89	55.57	28.70	16.15
Sugar, g	138.92	64.79	135.15	64.36	143.67	65.72
Retinol, *μ*g	544.16	580.03	574.34	741.92	506.10	264.88
Vitamin D, *μ*g	413.08	4190.69	2.10	1.69	931.27	6301.20
Vitamin E, mg	16.96	10.92	15.44	11.29	18.88	10.22
Vitamin K, *μ*g	224.29	809.37	369.69	1063.66	40.96	68.62
Sodium, mg	4186.21	1877.84	3704.17	1659.22	4793.99	1976.61
Vitamin B_6_, mg	3.39	6.10	2.78	1.10	4.17	9.09
Vitamin B_12_, mg	13.15	17.25	9.98	14.16	17.14	19.95
Folic acid, *μ*g	516.11	250.76	482.87	238.84	558.00	261.64
Potassium, mg	3955.15	1546.33	3661.51	1323.15	4325.39	1733.27
Calcium, mg	1064.89	446.12	995.13	374.87	1152.85	513.19
Magnesium, mg	396.63	146.80	380.06	151.26	417.51	139.81
Phosphorus, mg	1572.13	668.12	1486.98	736.21	1679.50	560.39
Iron, mg	12.87	5.03	11.79	4.71	14.21	5.16

MUFA: monounsaturated fatty acids; PUFA: polyunsaturated fatty acids; SFA: saturated fatty acids; SD: standard deviation.

**Table 3:** Food intake of the study sample.

	Total (*n* = 104)		Women (*n* = 58)		Men (*n* = 46)	
	Mean	SD	Mean	SD	Mean	SD
Shawarma	9.24	13.77	7.24	12.69	11.76	14.77
Falafel	6.85	11.91	6.20	14.49	7.66	7.56
Hamburger	3.88	11.25	4.06	11.61	3.63	10.91
Pizza and pies	18.50	37.67	9.99	15.46	29.23	52.29
Desserts	50.84	49.47	54.11	49.95	46.72	49.08
Traditional Arab sweets	17.03	28.72	15.91	30.85	18.43	26.06
Mayonnaise	1.92	3.29	1.26	2.25	2.73	4.13
Sucrose	21.05	17.94	17.58	16.11	25.41	19.30
Salt	4.15	2.50	3.65	1.80	4.77	3.08
Butter	7.75	10.58	7.11	10.21	8.56	11.08
Alcoholic beverages	1.27	6.32	1.79	8.06	0.61	2.89
Carbonated beverages	40.50	87.21	31.63	86.85	51.67	87.32
Olives	43.47	44.68	43.39	45.88	43.57	43.62
Fruits	316.45	216.41	351.84	221.09	271.83	204.03
Grains	211.47	114.79	177.74	91.17	254.00	127.74
Eggs	18.55	19.77	18.41	22.49	18.72	15.93
Vegetable oils	43.85	31.58	35.24	17.97	54.71	40.71
Nuts and dried fruits	37.22	45.02	39.21	48.78	34.70	40.18
Vegetables	382.99	271.95	353.98	247.74	419.57	298.47
Chicken	37.57	35.03	34.62	37.51	41.28	31.63
Meat	36.57	37.48	31.72	31.24	42.67	43.72
Fish	6.28	12.74	5.29	10.47	7.52	15.16

All foods are presented in grams.

SD: standard deviation.

**Table 4:** Comparison of nutrient intake by patients with different number of metabolic disorders.

	No MetS component		One MetS component		Two MetS components		Three MetS components		*p* value
	Media	SE	Media	SE	Media	SE	Media	SE	
Energy, Kcal	2200.4	242.4	2214.1	150.4	2693.9	194.6	2700.1	140.4	0.07
Carbohydrates, g	288.2	30.7	275.4	26.9	330.8	26.0	313.9	17.8	0.5
Fiber, g	19.0	2.9	19.6	1.7	21.6	2.3	22.7	1.6	0.5
Protein, g	75.9	10.0	71.4	5.5	92.6∗	7.3	92.2∗	4.9	0.03
Dietary cholesterol, mg	345.7	56.5	278.8	26.4	265.5	30.9	326.0	23.9	0.3
Fat, g	91.7	10.9	93.4	7.4	119.6	10.9	133.5∗	10.0	0.01
MUFA, g	38.1	5.4	38.8	3.3	53.4	5.2	57.5∗	5.1	0.01
PUFA, g	14.7	2.3	17.3	1.9	23.1	2.8	24.3∗	2.4	0.02
SFA, g	22.9	2.6	23.0	3.2	27.0	3.2	40.2	10.1	0.3
Sugar, g	144.7	22.5	139.7	13.1	125.8	10.6	147.7	10.9	0.5
Retinol, *μ*g	467.9	73.9	505.0	47.7	434.3	49.3	670.3	134.3	0.3
Vitamin D, *μ*g	2.7	0.7	2.0	0.2	1.8	0.3	2.5	0.3	0.2
Vitamin E, mg	12.1	1.8	14.0	1.1	17.2	2.1	19.4∗	1.9	0.04
Vitamin K, *μ*g	56.4	39.1	273.6	242.2	243.7	148.3	225.6	114.4	0.3
Sodium, mg	3723.6	371.0	3629.9	233.8	4243.1	339.8	4524.7	344.8	0.2
Vitamin B_6_, mg	3.1	0.5	2.7	0.2	2.7	0.2	4.4	1.5	0.6
Vitamin B_12_, mg	6.6	2.1	18.3	4.2	12.8	3.2	12.5	2.6	0.06
Folic acid, *μ*g	640.0	96.7	440.1	44.7	440.1	34.8	584.2∗	42.4	0.03
Potassium, mg	3963.3	577.8	3368.1	300.4	3901.6	300.9	4282.7	213.8	0.1
Calcium, mg	1200.3	183.9	962.0	75.6	1002.4	73.0	1132.4	73.8	0.3
Magnesium, mg	344.7	37.2	358.8	23.0	403.6	27.8	422.2	24.6	0.2
Phosphorus, mg	1486.5	221.5	1467.6	165.4	1580.3	137.8	1637.4	80.6	0.8
Iron, mg	11.0	1.4	11.7	1.0	13.1	0.9	13.7	0.8	0.3

MUFA: monounsaturated fatty acids; PUFA: polyunsaturated fatty acids; SFA: saturated fatty acids; SE: standard error. Data compared by one-way ANOVA. ^∗^Statistically significant difference (*p* < 0.05) from the group without any metabolic alteration by the post hoc Dunnett's test.

**Table 5:** Results of the principal component analysis of the nutritional intake.

	Component 1	Component 2	Component 3	Component 4	Component 5
Energy	**0.286**	0.041	0.156	0.089	0.083
Fiber	**0.245**	0.243	0.111	−0.158	−0.004
Protein	**0.252**	0.073	−0.122	−0.032	0.121
Dietary cholesterol	**0.163**	−0.236	−0.426	0.078	0.020
Fat	**0.253**	−0.265	0.145	0.169	0.080
Retinol	**0.139**	−0.173	−0.214	−0.272	−0.270
Vitamin D	**0.158**	−0.209	−0.208	−0.359	−0.161
Vitamin B_12_	**0.121**	−0.064	0.084	−0.511	−0.194
Potassium	**0.251**	0.202	−0.068	−0.124	−0.050
Carbohydrates	0.219	**0.348**	0.170	0.038	0.042
Sugar	0.216	**0.252**	0.194	0.085	−0.160
Vitamin K	0.008	**0.257**	0.226	0.087	−0.632
MUFA	0.227	−0.266	**0.251**	0.213	0.123
PUFA	0.218	−0.313	**0.272**	0.078	0.063
SFA	0.050	−0.199	**0.166**	−0.436	0.095
Vitamin E	0.234	−0.256	**0.254**	0.051	0.004
Folic acid	0.211	−0.030	−0.242	**0.323**	−0.211
Sodium	0.212	0.024	−0.128	0.042	**0.217**
Vitamin B_6_	0.070	0.358	−0.197	−0.126	**0.477**

MUFA: monounsaturated fatty acids; PUFA: polyunsaturated fatty acids; SFA: saturated fatty acids. The bold values are the loadings of each variable correlated to each component. The values are in order to the association for each component.

**Table 6:** Results of the factorial analysis. Loads of each dietary pattern detected in the factorial analysis.

	Dietetic pattern 1	Dietetic pattern 2	Dietetic pattern 3	Dietetic pattern 4	Dietetic pattern 5
Carbonated beverages	**0.623**			0.218	0.125
Grains	**0.323**	−0.126		0.108	
Chicken	**0.751**	0.429		0.206	−0.172
Meat	**0.554**	0.121			
Falafel		**0.730**			0.186
Olives	−0.251	**0.263**		0.149	0.126
Fruits		**0.378**			0.120
Eggs	0.162	**0.568**		0.180	−0.153
Vegetables		**0.453**			0.199
Desserts			**0.425**		0.122
Traditional Arabic sweets	0.257		**0.928**	-0.104	−0.233
Sucrose		−0.221	**0.287**		0.184
Butter	−0.102		**0.266**	0.182	
Vegetable oils	0.253	0.128	0.180	**0.295**	0.181
Shawarma	0.116			**0.261**	−0.126
Mayonnaise	0.142		0.180	**0.966**	
Fish		0.192		**0.201**	0.131
Hamburger					**0.109**
Pizza and pies		0.158			**0.324**
Salt	0.269		0.195		**0.708**
Nuts and dried fruits		0.168	0.205		**0.328**
	24	23	20	18	15

Percentage of variance explained by each dietary pattern. The bold values are the loadings of each variable correlated to each dietetic pattern. The values are in order to the association for each dietetic pattern.

**Table 7:** Linear regression analysis between metabolic syndrome components and main components of nutritional intake and dietary patterns.

	Hyperglycemia		Hypertriglyceridemia		Hypercholesterolemia		High LDL		Low HDL		High SBP		High DBP	
	*β* coefficient	*p* value	*β* coefficient	*p* value	*β* coefficient	*p* value	*β* coefficient	*p* value	*β* coefficient	*p* value	*β* coefficient	*p* value	*β* coefficient	*p* value
*Components of nutritional intake*														
Component 1	−1.52	0.09	0.36	0.9	−0.27	0.8	18.89	0.6	0.05	0.9	0.79	0.04	0.64	0.02
Component 2	−1.16	0.6	−7.13	0.1	−0.59	0.8	2.24	0.4	0.02	0.9	−1.65	0.06	−0.73	0.3
Component 3	4.01	0.08	3.83	0.4	1.80	0.5	−4.46	0.1	−1.37	0.2	−0.45	0.6	−0.46	0.5
Component 4	1.39	0.6	−4.29	0.4	−0.60	0.84	−3.61	0.3	0.67	0.6	−0.07	0.9	−0.42	0.6
Component 5	3.25	0.3	5.16	0.4	3.20	0.4	−1.89	0.6	−1.23	0.4	−1.38	0.3	−1.11	0.2

*Dietetic pattern*														
Pattern 1	11.70	0.5	130.92	0.001	39.52	0.08	8.21	0.8	−5.53	0.5	−2.14	0.8	2.06	0.7
Pattern 2	−8.59	0.6	−31.43	0.3	−6.12	0.7	23.20	0.5	−5.58	0.4	−1.57	0.8	5.26	0.3
Pattern 3	58.06	0.01	32.35	0.5	−40.05	0.2	14.75	0.6	6.14	0.6	10.65	0.3	12.52	0.09
Pattern 4	−5.69	0.8	18.50	0.7	−9.98	0.7	13.15	0.6	−7.68	0.4	4.54	0.6	12.5	0.06
Pattern 5	6.47	0.8	94.2	0.05	−15.76	0.6	3.10	0.9	10.72	0.4	2.54	0.8	0.05	0.9

All models were adjusted for sex and age, and independent variables were introduced to the model in a quantitative form. *β*: regression coefficient.

**Table 8:** Risk of metabolic syndrome components based on the components of nutritional intake and dietary pattern.

	Hyperglycemia		Hypertriglyceridemia		Hypercholesterolemia		High LDL		Low HDL		Hypertension		Metabolic syndrome	
	OR (95% CI)	*p* value	OR (95% CI)	*p* value	OR (95% CI)	*p* value	OR (95% CI)	*p* value	OR (95% CI)	*p* value	OR (95% CI)	*p* value	OR (95% CI)	*p* value
*Components of nutritional intake*														
Component 1	0.39 (0.81–1.09)	0.4	1.02 (0.88–1.17)	0.8	1.02 (0.90–1.14)	0.8	1.07 (0.92–1.25)	0.4	0.96 (0.84–1.09)	0.5	1.16 (0.99–1.36)	0.06	1.06 (0.93–1.22)	0.4
Component 2	1.19 (0.85–1.66)	0.3	0.77 (0.53–1.12)	0.2	1.24 (0.91–1.70)	0.2	1.39 (0.97–2.01)	0.07	1.04 (0.77–1.38)	0.9	0.58 (0.36–1.01)	0.05	1.04 (0.75–1.44)	0.8
Component 3	1.22 (0.84–1.79)	0.3	1.03 (0.72–1.48)	0.8	1.15 (0.82–1.60)	0.4	0.74 (0.48–1.13)	0.2	1.20 (0.86–1.69)	0.3	0.85 (0.56–1.28)	0.4	1.06 (0.73–1.56)	0.8
Component 4	0.98 (0.65–1.49)	0.9	0.97 (0.66–1.42)	0.8	0.83 (0.58–1.18)	0.3	0.72 (0.48–1.06)	0.09	0.94 (0.66–1.33)	0.7	1.27 (0.76–2.15)	0.4	1.10 (0.71–1.71)	0.7
Component 5	1.46 (0.84–2.58)	0.2	1.04 (0.66–1.63)	0.9	0.97 (0.65–1.46)	0.9	1.05 (0.64–1.72)	0.9	1.75 (1.00–3.04)	0.05	0.88 (0.53–1.49)	0.7	1.55 (0.88–2.73)	0.1

*Dietetic pattern*														
Pattern 1	4.32 (0.24–77.5)	0.3	13.6 (0.78–235)	0.07	5.05 (0.31–81.9)	0.2	31.7 (1.31–773)	0.03	9.05 (0.56–146.3)	0.1	1.12 (0.03–38.5)	0.9	47.1 (2.00–111)	0.02
Pattern 2	1.05 (0.11–10.3)	0.9	0.22 (0.02–3.33)	0.2	0.85 (0.10–7.02)	0.9	0.32 (0.02–6.61)	0.4	1.34 (0.17–10.5)	0.8	8.14 (0.79–84.4)	0.08	1.28 (0.13–12.9)	0.8
Pattern 3	4.26 (1.55–11.7)	0.005	1.94 (0.05–66.4)	0.7	0.32 (0.01–8.92)	0.5	13.6 (0.26–720)	0.2	0.38 (0.01–11.7)	0.6	5.29 (0.10–277)	0.4	0.07 (0.01–4.79)	0.2
Pattern 4	0.38 (0.01–6.01)	0.4	3.43 (0.13–91.1)	0.5	0.65 (0.04–11.7)	0.8	1.79 (0.05–65.5)	0.8	7.10 (0.39–170)	0.2	11.9 (0.22–629)	0.2	0.28 (0.01–7.11)	0.4
Pattern 5	4.53 (0.09–217)	0.4	0.11 (0.01–3.72)	0.2	0.41 (0.02–10.9)	0.6	0.51 (0.01–23.9)	0.7	0.33 (0.01–8.64)	0.5	3.88 (0.05–282)	0.5	0.17 (0.01–6.09)	0.3

All models were adjusted for sex and age, and independent variables were introduced to the model in a quantitative form. OR: odds ratio, 95% CI: 95% confidence interval.

## Data Availability

Data are available upon reasonable requests.

## Supplementary Materials

Supplementary Materials Supplementary Table S1: The description of the main drinks and dishes of Syrian food; in it, you can see the food groups, examples of each food, and the description of the food groups.
