# Supplementary material for: Dietary Patterns and Their Association with Metabolic Syndrome and Their Components in Middle-Class Adults from Damascus, Syria: A Cross-Sectional Study
Source: J Nutr Metab. 2022 Mar 24;2022:5621701. doi: 10.1155/2022/5621701 (PMC8970872; doi:10.1155/2022/5621701)
Supplement: Supplementary Materials — Supplementary Table S1: The description of the main drinks and dishes of Syrian food; in it, you can see the food groups, examples of each food, and the description of the food groups. [file 5621701.f1.docx]

Table S1. Description of the main drinks and dishes of Syrian food

| Food group | Example | Description |
| --- | --- | --- |
| Alcoholic beverages | Arak | The strongest of the anise-flavored liqueurs and has no added sugar. |
| Nonalcoholic beverages | Gaseous drinks  Orange juice, lemon with mint juice (polo) | Carbonated drinks or fizzy drinks  Fruit juices with sugar |
| Meat-based dishes | Kibbeh  Kebab  Kibbeh with yogurt  Shish tawook  Safiha | Minced lamb meat with bulgur and spices  Grilled spicy soft meat)  Fresh Kibbeh cooked with yoghurt  Grilled spicy small pieces of chicken  Baked paste of wheat filled with meat, yoghurt and onion |
| Cereal-based dishes | Keshke  Fatayer and borak  Kaa´k  Fette of chiken  Fette of eggplan  Rice with meat and bean  Rice with meat and peas  Rice with lentils | Bulgur and yogurt-based starter  Baked paste if wheat filed with spinach or cheese  Toasted bread paste  Arabic bread mixed with chicken stock, yogurt, onion and chicken pieces  Arabic bread mixed with yoghurt, tomato sauce and eggplant filled with meat and cooked with tomato sauce  Eaten with yogurt  Eaten with yogurt  Eaten with yogurt |
| Legumes based dishes | Falafel  Fette of chickpeas  Humus  Houboub (eaten in wintertime) | Fried triturated chickpeas and beans mixed with spices  Arabic bread mixed with yogurt and chickpeas  Puree made from ground sesame seeds  Beans, chickpeas and wheat cooked with water and sugar |
| Vegetable-based dishes | Yabrak  Eggplant mahshi  Zucchini mahshi  Taboule  Fattoush | Zucchini Mahshi  Stuffed eggplant  Stuffed zucchini  Vegetarian arabic salad  Salad with various diced vegetables mixed with jubz-type flatbread cut into strips or squares and fried |
| Dressings and accompaniments | Labaneh  Mutabal  Tahini | Cream cheese with mint  Eggplant dip mixed with salt, pepper, olive oil, tahini and pomegranate seeds  Paste made from ground sesame seeds |
| Fast food | Shawarma | Sandwich made of pita bread and stuffed with meat, chicken or vegetables |
| Sweets and cupcakes | Halawe  Konafe  Harise  Mamool  Kol wa shkor | Sesame oil cooked with sugar  Triturated wheat cooked with margarine and cheese soaked with katr (water cooked with sugar)  Triturated wheat cooked with margarine and katr covered with nuts  Paste of wheat mixed with margarine and sugar filled with pistachio or dates or walnut  Puff pastry soaked with katr and honey and filled with pistachio |
| Most consumed fruits | Orange, mandarin, banana, strawberries, grapes, apricot, watermelon, apple, peach, pear, grape fruit |  |
| Most consumed vegetables | Potatoes, tomato, spinach, eggplants, mouloukie, bamie, garlic, onion, pumpkin, cucumber, zucchini, beans, peas, carrots, capsicum, cabbage |  |
